# Cytological diagnosis of a rare case of solid pseudopapillary neoplasm of the pancreas

**DOI:** 10.4103/0970-9371.66693

**Published:** 2010-01

**Authors:** Kirana Pailoor, Hema Kini, Aarthi R. Rau, Yogesh Kumar

**Affiliations:** Department of Pathology, Kasturba Medical College, Light House Hill Road, Mangalore, India; 1 Department of Surgery, Kasturba Medical College, Light House Hill Road, Mangalore, India

**Keywords:** Solid and cystic tumor, pancreas, solid pseudopapillary neoplasm

## Abstract

A 23-year-old woman presented to our hospital with nonspecific pain in the abdomen. She underwent radiological investigations, which revealed a solid and cystic mass in the tail end of the pancreas. The mass was diagnosed to be solid pseudopapillary neoplasm of the pancreas on intraoperative scrape cytology. This was further confirmed by histopathology. The cytological diagnosis enabled appropriate surgical treatment to be planned and carried out without undue delay. It is important to distinguish this rare tumor from other pancreatic tumors with similar cytohistologic features because, if diagnosed correctly and managed surgically, this neoplasm is associated with a good prognosis.

## Introduction

Solid and cystic papillary epithelial neoplasm of the pancreas (SPENP) was first described by Frantz in 1959 and hence was also called the Gruber-Frantz tumor.[1] It is a distinctive and rare tumor, which accounts for 0.17–2.7% of all nonendocrine tumors of the pancreas and, to date, there are very few case reports on the cytological diagnosis of this tumor.[1–9] It occurs in adolescent girls and young women, more frequently in the body and tail of the pancreas. Because of its gross and cytohistologic features, it has been variously designated as solid and papillary epithelial neoplasm, solid and cystic tumor, solid pseudopapillary tumor of the pancreas, papillary and cystic neoplasm, papillary epithelial neoplasm and papillary epithelial tumor.[1,2] It forms an abdominal mass with nonspecific digestive symptoms. Although local invasion and infiltration of the capsule may occur, distant metastases is uncommon.[3]

## Case Report

A 23-year-old woman presented with a two-day history of mid-epigastric and periumbilical pain and also bilious vomiting. The pain was “crampy”, nonshifting and constant. She had periumbilical tenderness on palpation but did not have any rebound pain or guarding. She did not have any associated weight loss, fever, chills, malena, hematemesis, hematochezia or steatorrhoea. Physical examination revealed a deep-seated tender mass in the left upper quadrant.

An ultrasound of the abdomen and computed tomography (CT) scan revealed a well-circumscribed, solid mass in the tail of the pancreas measuring 4.6 × 4.7 cm with multiple tiny cysts, which extended up to the splenic hilum. A differential diagnosis of pancreatic endocrine neoplasm, solid pseudopapillary neoplasm and pancreatic carcinoma was considered.

**Figure 1:**
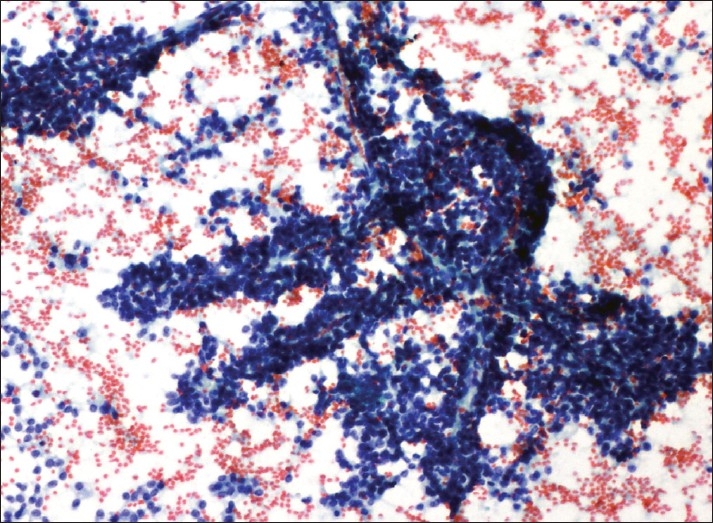
Cellular smear showing branching papillary fronds (Pap, ×100)

**Figure 2:**
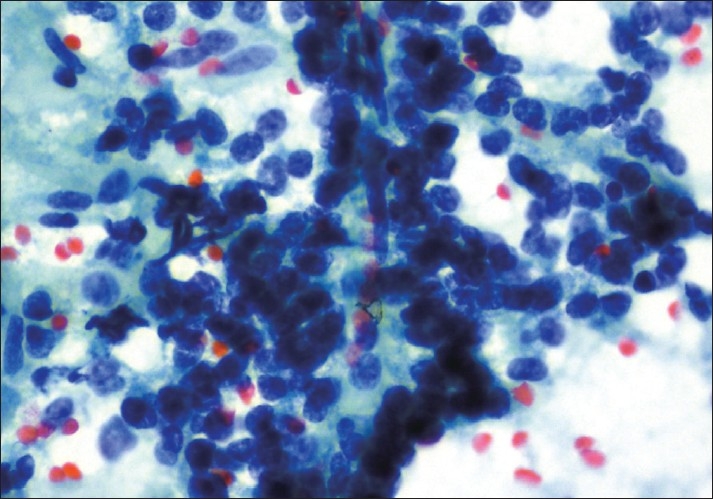
Cells have round to oval nuclei with fine granular chromatin and have nuclear grooves (Pap, ×400)

A laparotomy was performed and the tumor in the tail of the pancreas along with the spleen was excised. Scrape smears were prepared and stained using rapid hematoxylin and eosin and papanicoloau (PAP) method for cytological evaluation at intra-operative consultation. Highly cellular smears showed numerous delicate branching papillary fronds composed of fibrovascular stalks lined by one to several layers of uniform tumor cells [Figure 1]. These tumor cells were also arranged in small scattered clusters and dispersed singly. Cells were uniform with round to oval nuclei, with evenly dispersed, homogenous, finely granular chromatin and one to two small but distinct nucleoli. The delicate nuclear membrane showed frequent infoldings imparting a “coffee-bean” appearance. The cytoplasm was variable in amount and generally poorly defined. The background showed granular debris and foamy histiocytes [Figure 2]. A cytological diagnosis of solid pseudopapillary neoplasm of the pancreas was made.

The resected specimen consisted of the tail of the pancreas, with the tumor and the spleen. The histological features were similar to that of the cytology, consisting of monomorphic cells with ovoid nuclei, fine chromatin, some with nuclear grooving and clear to eosinophilic cytoplasm, arranged in a solid, pseudopapillary, microcystic and trabecular pattern. Amidst the tumor, numerous thin-walled vessels, bands of fibrous tissue, coagulative necrosis and hemorrhage were seen. Also seen were nests of tumor cells extending into the capsule. These features confirmed the cytological diagnosis of solid pseudopapillary tumor of the pancreas. Periodic acid Schiff stain was carried out, which was negative for glycogen, and confirmed our diagnosis.

## Discussion

Cystic tumors of the pancreas account for only 6% of exocrine tumors. The various entities in this group occur more frequently in females, but differ in age at presentation. It is important to distinguish SPENP from other pancreatic masses such as mucinous tumors, microcystic adenoma, ductal adenocarcinoma, acinar cell carcinoma and islet cell tumors to enable appropriate surgical treatment because this neoplasm is associated with a good prognosis.[4] A few of these lesions have been diagnosed by preoperative percutaneous fine needle aspiration and intraoperative scrape cytology.[2,6] The branching papillary fronds with monomorphic tumor cells having a palisaded appearance around a fibrovascular core, lack of pleomorphism and mitoses, round to oval nuclei with fine chromatin distribution and occasional nuclear folding are characteristic of SPENP. Eosinophilic mucoid globules that appear red on May-Grünwald-Giemsa (MGG) stain are also characteristic features of this tumor.[2,7,8] However, MGG stain was not performed in our case. Mucinous tumors occur between 20 and 60 years, with a thick mucoid aspirate. The cells are columnar with eccentric nuclei and clear cytoplasm with abundant extracellular mucoid material in the background. These cells may be bland and uniform or cytologically malignant.[4] Microcystic adenoma occurs at an older age (61–68 years), with watery aspirate and scanty tumor cells with clear cytoplasm, small round nuclei and inconspicuous nucleoli.[4] A positive glycogen stain easily differentiates microcystic adenoma from SPENP, which lacks glycogen, as in our case. Acinar cell carcinoma shows acinar and glandular structures. Aspirates from ductal adenocarcinoma show three-dimensional cell clusters and occasional papillae. The tumor cells usually show obvious features of malignancy.[4] Islet cell tumors are rare and show monomorphic cells that may form perivascular aggregates and nuclei with salt and pepper chromatin.[4] Electron microscopy is of great value in achieving a correct diagnosis of SPENP. The cells have eccentrically placed nuclei with an indented contour and occasional nucleoli. The cytoplasm contains numerous mitochondria. Cell junctions, mostly primitive, have been noted, but true desmosome formation is rare. Evidence of both exocrine and endocrine differentiation has been described, with exocrine-type cells containing larger, dense, membrane-bound zymogen-type granules and endocrine-type cells containing smaller neurosecretory granules.[9]

Although the histogenesis of this tumor is uncertain, SPENP is thought to originate from the epithelium of the smallest ductules in the pancreas, which are believed to harbor multipotential cells throughout life.[6] Other authors believe that it may arise from a totipotential stem cell with endocrine or exocrine or dual differentiation.[4] Microscopically, these tumors have a mixture of solid, cystic and pseudopapillary patterns.

Cells are uniform and polygonal with clear to eosinophilic abundant cytoplasm, which is glycogen and mucin negative.[7] Mitotic figures are rare. Immunohistochemically, there is reactivity for α_1_-antitrypsin, CD56, vimentin and beta-catenin.[5] Another marker consistently expressed, and of diagnostic utility, is CD10.[8] Progesterone receptors have been detected both immunohistochemically and by standard biochemical methods, which suggests a hormone-dependent neoplasm.[8]

Solid pseudopapillary neoplasm of the pancreas is a distinct clinicopathological entity and is associated with good prognosis. Recognition of the cytological features of this tumor, both preoperatively and intraoperatively, is valuable in planning appropriate surgical management of these patients.

## References

[1] Pettinato G, Manivel JC, Ravetto C, Terracciano LM, Gould EW, di Tuoro A (1992). Papillary cystic tumor of the pancreas. A clinicopathologic study of 20 cases with cytologic, immunohistochemical, ultrastructural, and flow cytometric observations, and a review of the literature. Am J Clin Pathol.

[2] Katz LB, Ehya H (1990). Aspiration cytology of papillary cystic neoplasm of the pancreas. Am J Clin Pathol.

[3] Bondeson L, Bondeson AG, Genell S, Lindholm K, Thorstenson S (1984). Aspiration cytology of a rare solid and papillary epithelial neoplasm of the pancreas. Light and electron microscopic study of a case. Acta Cytol.

[4] Naresh KN, Borges AM, Chinoy RF, Soman CS, Krishnamurthy SC (1995). Solid and papillary epithelial neoplasm of the pancreas. Acta Cytol.

[5] Deshpande V, Gray W, Mckee GT (2003). Pancreas. Diagnostic cytopathology.

[6] Foote A, Simpson JS, Stewart RJ, Wakefield JS, Buchanan A, Gupta RK (1986). Diagnosis of the rare solid and papillary epithelial neoplasm of the pancreas by fine needle aspiration cytology. Light and electron microscopic study of a case. Acta Cytol.

[7] Thompson LDR, Heffess CS, Mills SE, Carter D, Greenson JK, Oberman HA, Reuter V, Stoler MH (2004). Pancreas. Sternberg’s diagnostic surgical pathology.

[8] Rosai J, Rosai J (2004). Pancreas and periampullary region. Ackerman’s surgical pathology.

[9] Jhala N, Siegal G P, Jhala D (2007). Fine needle aspiration – A powerful modality in the preoperative diagnosis of solid pseudopapillary neoplasm of the pancreas. Path Case Rev.

